# State of the Art on Functional Virgin Olive Oils Enriched with Bioactive Compounds and Their Properties

**DOI:** 10.3390/ijms18030668

**Published:** 2017-03-20

**Authors:** Patricia Reboredo-Rodríguez, María Figueiredo-González, Carmen González-Barreiro, Jesús Simal-Gándara, María Desamparados Salvador, Beatriz Cancho-Grande, Giuseppe Fregapane

**Affiliations:** 1 Nutrition and Bromatology Group, Analytical and Food Chemistry Department, Faculty of Science, University of Vigo, Ourense Campus, E-32004 Ourense, Spain; preboredo@uvigo.es (P.R.-R.); mariafigueiredo@uvigo.es (M.F.-G.); cargb@uvigo.es (C.G.-B.); jsimal@uvigo.es (J.S.-G.); 2 Food Technology Department, Faculty of Chemistry, University of Castilla-La Mancha, Ciudad Real Campus, E-13071 Ciudad Real, Spain; Amparo.Salvador@uclm.es

**Keywords:** enriched olive oil, functional food, phenolic compounds, health, oxidative stress, endothelial dysfunction, intestinal immune function

## Abstract

Virgin olive oil, the main fat of the Mediterranean diet, is per se considered as a functional food—as stated by the European Food Safety Authority (EFSA)—due to its content in healthy compounds. The daily intake of endogenous bioactive phenolics from virgin olive oil is variable due to the influence of multiple agronomic and technological factors. Thus, a good strategy to ensure an optimal intake of polyphenols through habitual diet would be to produce enriched virgin olive oil with well-known bioactive polyphenols. Different sources of natural biological active substances can be potentially used to enrich virgin olive oil (e.g., raw materials derived from the same olive tree, mainly olive leaves and pomaces, and/or other compounds from plants and vegetables, mainly herbs and spices). The development of these functional olive oils may help in prevention of chronic diseases (such as cardiovascular diseases, immune frailty, ageing disorders and degenerative diseases) and improving the quality of life for many consumers reducing health care costs. In the present review, the most relevant scientific information related to the development of enriched virgin olive oil and their positive human health effects has been collected and discussed.

## 1. Market Needs for Functional Oils

The tenet “let food be thy medicine and medicine be thy food” was postulated by Hippocrates 2500 years ago. Therefore, it was established that foods provide benefits to our health. This concept was first developed in Japan in the 1980s when, faced with escalating health care costs, the Ministry of Health and Welfare initiated a regulatory system to approve foods called Foods for Specified Health Use (FOSHU), with documented health benefits in hopes of improving the health of the nation’s aging population [1]. In spite of the fact that Europe and United States governments do not have a formal definition for “functional food”, European Union researchers concluded that “food products can only be considered functional if together with the basic nutritional impact it has beneficial effects on one or more functions of the human organism, thus either improving the general and physical conditions or/and decreasing the risk of the evolution of diseases” [1].

Consumers are taking greater responsibility for their own health and they are increasingly turning to their diet to improve it. In fact, the Mediterranean diet is characterized by a high intake of exogenous dietary phenolics as a consequence of a high intake of virgin olive oil (VOO), fruit, nuts, vegetables, and cereals; a moderate intake of fish and poultry; a low intake of dairy products, red meat, processed meats, and sweets; and wine in moderation, consumed with meals [2]. Results from the Prevention with Mediterranean Diet (PREDIMED) study showed that the higher phenolic intake of the Mediterranean diet is associated with a lower incidence of cardiovascular diseases, metabolic syndrome, cancer and age-related cognitive decline [3,4,5,6,7,8,9,10,11].

In the Mediterranean diet, olive oil (OO) is the main added lipid source used in the preparation of foods, both raw (e.g., in salads or condiments), as ingredient in recipes, as well as for cooking (e.g., frying or grilling). Its chemical composition consists of major compounds (representing more than 98% of the total oil weight) and minor compounds (about 2% of the total oil weight, including more than 230 chemical compounds, e.g., aliphatic and triterpenic alcohols, sterols, hydrocarbons, volatile compounds and antioxidants) [12]. The main antioxidants of VOO comprise carotenes and unique bioactive phenolic compounds (lipophilic and hydrophilic phenols). Even though lipophilic phenols (e.g., tocopherols) can be found in other vegetable oils, the hydrophilic phenolic composition is characteristic only of VOOs [12]. Hydroxytyrosol (HTyr/HT), mainly present as a secoiridoid derivative together with minor amounts of free forms and the acetylated derivative (HTyr acetate), is the most representative phenol among the hydrophilic compounds in VOO followed by tyrosol (Tyr) and its secoiridoid derivatives. In addition to phenolic alcohols, other chemical phenolic groups such as flavones, lignanes and isochromans can be found in minor extent.

Several bioavailability studies have confirmed that polyphenols are dose-dependently absorbed and in addition, a high intake of olive oil phenols increases their plasma concentrations and excretion [13,14,15]. Rich-polyphenol VOO has been shown to improve antioxidant and anti-inflammatory effects and to reduce the proliferation of cell adhesion molecules compared with low-polyphenol VOO [16,17,18,19,20]. Indeed, the Effect of Olive Oil on Oxidative Damage on European Populations (EUROLIVE) study highlighted an increase in high-density lipoprotein cholesterol (HDL-C) levels, a decrease of in vivo lipid oxidative damage and an increase in HDL cholesterol efflux from macrophages [14,21,22] in a direct relationship with the phenolic compounds (PC) of olive oil. The same results on VOO intake in healthy humans were firstly described by Hernáez et al., 2014 [23]. Consequently, the European Food Safety Authority (EFSA) approved a health claim on olive oil polyphenols [24]. This health claim established literally that “olive oil polyphenols contribute to the protection of blood lipids from oxidative stress and also stated that the claim may be used only for olive oil containing at least 5 mg of HTyr and its derivatives (e.g., oleuropein complex and Tyr) per 20 g of olive oil; in order to bear the claim information shall be given to the consumer that the beneficial effect is obtained with a daily intake of 20 g of olive oil”. Olive oil PC have also positive effects on other physiological parameters such as inflammatory markers, platelet and cellular function and cardiovascular health [8,25,26,27,28,29,30].

The daily intake of endogenous bioactive phenolics from VOO is variable due the influence of multiple agronomic and technological factors, being the cultivar and the ripening index some of the most important ones. Therefore, a large variety of VOO can be found in the market, containing not only quite different total polyphenol content but also quite different polyphenol compositions [31]. Thus, a good strategy to ensure an optimal intake of polyphenols through habitual diet would be to produce enriched VOO (En-VOO) with well-known bioactive polyphenols [32,33,34,35,36,37,38,39].

By 2035, 70 million people will be aged >65 years old. The large increase of the aged population will imply a greater incidence of chronic diseases of aging such as heart disease, cancer, osteoporosis, Alzheimer’s disease and age-related macular degeneration, among others, and will involve a high health cost. In fact, the prevention of pathologies by diet is an important public health challenge in order to reduce the morbidity and mortality and the cost to society. In this sense, En-VOO could contribute to promote health while maintaining or reducing diet fat content.

In the present review, for the first time, the most relevant scientific information related to the development of En-VOO and their positive human health effects is collected and discussed.

## 2. Sources of Natural Bioactive Ingredients to Enrich Oils

A great number of different sources of natural biological active substances, also known as functional, can be potentially used to enrich VOO [38]. Several published studies on VOO enrichment employed raw materials derived from the same olive tree, mainly leaves or residual olive pomace obtained after the mechanical extraction of the oil, being a cheap and good source of phenolic compounds to be used to increase their concentration in the developed enriched oil. Other studies used plants and vegetables, mainly herbs and spices. These sources of natural bioactive ingredients employed to develop novel functional oils are summarized in Table 1 and briefly discussed below.

***Olive leaves*.** An organic-solvent free extraction to yield phenolic compounds from the olive leaves has been reported [40,41], using ultrasound or microwave-assisted extraction (MAE) in order to speed up the process. As expected, the En-VOO experienced a noticeable improvement in their quality stability parameters (e.g., peroxide value (PV) or Rancimat induction period) as compared with their non-enriched counter parts, depending on the final concentration in phenolic compounds [40,41,42,43]. In other cases, the enrichment of olive oil, or other edible oils, with leaf extracts has been proposed to produce a more stable frying oil [44,45], moreover, the content in phenolic compounds in the fried product, potatoes, was also increased, in this specific case, with the corresponding added nutrition value.

***Olive pomace*.** Since the phenolic compounds in the olive’s past—that have been chemically transformed through the milling process, basically hydrolysed from their glucoside form—are mainly polar, only a small amount is solubilized in the oil during the malaxation operation [46]. Consequently, most phenolics are found in the residual byproduct olive pomace (95%–98% of the olive fruit content), which is therefore another fine and cheap source of these bioactive compounds [38,42].

Similar extraction procedures, as for the case of leaf extracts, were employed to obtain olive pomace extracts; furthermore, the use of an accelerated extraction system (ASE) was also reported [42]. In Section 3 of this article, a discussion on the different methods and procedures used to enrich VOO is described. In several cases, with the purpose of studying the specific effect of the spiked phenolics, a refined olive oil has been used as control, because is practically free of its original phenolics. 

***Olive oil extracts*.** In a few cases, the same phenolic compounds contained in the VOO are extracted and then used to enriched the own VOO [49], to allow the preparation of oil samples with a greater and well known amount and type of phenolic compounds. Furthermore, instead of the addition of phenolic extracts, it is also feasible to enrich the VOO by employing appropriate technological parameters during processing, mainly the intensity of milling and the temperature–time conditions of the malaxation [50,71].

***Herbs and spices*.** Apart from olive leaves and pomace, there are many other sources of bioactive compounds from plants and vegetables, mainly herbs and spices, very commonly used in gastronomy for millennia by means of infusion or maceration process. Nowadays, the market for these products has gained more interest because of their sensory properties, which are perceived by consumers as foodstuffs with an added value. Indeed, spices and herbs are traditionally added to olive oil in Mediterranean cuisine to enhance its aroma and taste [72]. Besides affecting the oil’s sensory characteristics, the presence of herbs and spices has an impact on the nutritional value of the flavoured oils, due to their content in natural biological active families of compounds. 

Many herbs and spices have been studied to modify the flavour and/or increase the content in bioactive compounds in VOO:

- Red pepper [51,52]
- Hot pepper, garlic, oregano and rosemary [53]
- Rosemary, lavender, sage, menthe, basil, lemon and thyme [54]
- Garlic, lemon, oregano, hot pepper, and rosemary [55]
- Basil [56]
- Lemon and thyme [57]
- Thyme [36]
- Oregano [58,59]
- Thyme [60]
- Garlic, hot chili peppers, laurel, oregano and pepper [61].
- Sweet lemon and sweet orange peels [62]
- Basil, chili and garlic [63]
- Caraway [64]
- Thyme and oregano [65]

The flavouring techniques with herbs and spice extracts influenced the chemical composition and sensory characteristics of these novel oils significantly. Concerning the stability and antioxidant capacity of the En-VOO, the published results are still quite contradictory, mainly due to the different experimental conditions employed (such as the extraction method, the concentration of the added extract, the type of vegetable raw material, and the stability and antioxidant capacity assays carried out). In general, the flavouring of VOO with herb and spice extracts does not clearly improve the stability (Rancimat or oxidative stability index (OSI) induction period) or the apparent evolution of the oxidation indices (i.e., PV; specific UV extinction coefficients (K_232_ and K_270_); or hexanal content) of the En-VOO. Nevertheless, in several cases its antioxidant capacity—measured by the 2,2-diphenyl-1-picrylhydrazyl (DPPH), oxygen radical absorbance capacity (ORAC) or 2,2′-azino-bis(3-ethylbenzothiazoline-6-sulphonic acid) (ABTS) assays—does increase due to higher amount and also the different activity of the specific bioactive compounds from herbs and spices as compared to those from VOO. Some particular cases are discussed below:

It has been reported the addition of red pepper extracts, obtained by supercritical fluid extraction (SFE), at low extraction pressure (e.g., 16.2 MPa) and containing low capsaicinoid levels, did not affect olive oil stability. However, a slight pro oxidant effect was observed when the *Capsicum frutescens* was extracted at higher pressure [52]. Similarly, dried chili pepper infusion significantly enriched olive oil with antioxidant compounds and also modified its volatile profile, nevertheless, its addition caused a significant increase in PV and hexanal, related to higher oxidation rate [51].

The enrichment of several aromatic plants (hot pepper, garlic, oregano and rosemary) improved the medium-term storage stability of the extra virgin olive oil (EVOO) [53]; since, at the end of the storage period studied (3–4 months), all samples of flavoured oils showed lower (PV and K_232_) or similar (acidity, K_270_ and (*E*)-2-hexenal/hexanal) values of oxidation indices than the control oil. Moreover, samples with added oregano showed lower lipid oxidation indicator values (K_232_, K_270_, peroxide and anisidine values), especially in darkness, increasing the shelf-life of the En-VOO [58]. In another study, the capability to counteract oxidation of oregano En-VOO was generally slightly improved (induction period of 9.4 h in control oil and 10.4 h with oregano), although practically, no differences in DPPH and ABTS tests were observed [61].

In another study, the ORAC assay showed that En-VOOs produced a substantial increase in their antioxidant capacity as compared to the control oil, which gradually increased with the thyme phenolic enrichment, due to the potent antioxidant capacity of this herb as reported for dried thyme (ranging from 131,000 to 139,400 μmol Trolox euivalent/100 g) [36].

However, in another work, except the garlic-flavoured olive oil, all the other enriched oils (lemon, oregano, hot pepper, and rosemary) presented a higher PV than control at 6 and 9 months storage time [55]. Moreover, during storage rosemary flavoured oil was the only one showing a higher antioxidant activity than VOO control (according to the β-carotene bleaching assay coefficients; i.e., 51.3 ± 4.2 at 9 months, as compared to 8.5 ± 0.7 for hot pepper, lemon, oregano, unflavoured, and garlic). Similarly, the oxidative stability of the flavoured product did not change while enriched with lemon and thyme extracts—although some bioactive compounds, such as limonene and carvacrol were transferred [57].

The addition of citrus zests increased the polyphenols and carotenoids contents of the flavoured oils, producing an increase in its antioxidant activity (by means of DPPH-radical scavenging activity and β-carotene‑linoleate bleaching assay); nevertheless, the control sample showed a lower PV and higher oxidative stability in all the exposure times tested in comparison with enriched samples, moreover the degradation rate of bioactive compounds was also lower for VOO comparing to flavoured oils [62].

Flavoured VOO with caraway showed that samples obtained by ultrasound-assisted extraction (USAE) presented the highest induction period (6.39 h) followed by conventional maceration (4.45 h) and the untreated sample (3.45 h). On the contrary, results indicate an increase in PV of conventionally aromatised oils (2.7 meq·O_2_/kg) in comparison to un-aromatised oil, as well as a slight increase in ultrasound-treated samples for the K_232_ and K_270_ oxidation indices [64].

In other cases, the effect on the sensory properties and consumers’ perception of the flavoured olive oil was the main purpose of the enrichment process [51,53,54,58,63,66,72]. As expected, organoleptic evaluation generally confirmed that the flavouring with vegetable materials during the VOO-making process impart sensory attributes which made this product significantly different from the original VOO employed. The practice of olive oil flavouring, leading to the so called “gourmet oils”, could therefore increase the use of olive oil among non-traditional consumers and, at the same time, add further value to this agricultural product.

***Other bioactive compounds of plant origin*.** Lycopene—a bioactive red pigment and the most potent in vitro antioxidant among carotenoids—is one of the most studied and indeed lycopene-enriched VOO is commercially available [35,70,73]. Its incorporation into the diet may enhance the health-promoting effects of the VOO, contributing as a functional tool against several disorders where oxidative stress plays an important role [35]. A patented method based on direct extraction of lycopene with the olive oil without using solvents or other harmful compounds has been also proposed [69].

Another example of a specific functional component is the enrichment with carnosic acid-aditerpene found in various *Lamiaceae* such as sage and rosemary, which is the most powerful antioxidant among diterpenes. Its antioxidant mechanism has been studied [74]. The results of oxidative stability obtained at 60 °C showed a dose-dependent inhibition in the formation of primary and secondary oxidation products and an enhancement of radical scavenging activity.

Functional or structured lipids—tailor-made fats and oils with improved nutritional or physical properties—have also been used to enrich VOO with the purpose of enhancing its health properties. VOO enriched with phospholipids (soy lecithin) up to the levels present in seed oils (from 2.5 to 10.0 g/kg) was studied as a potential functional food [75]. Furthermore, the production of olive oil enriched with medium chain fatty acids (MCFA) has also been reported [76].

## 3. Optimization of the Development of Novel Functional Oils

As stated before, the content and profile in phenolic compounds in the olive fruit depend on different agronomical parameters, viz soil, irrigation, climate, and so on, but mainly on the variety of the olive employed [77], as well as technological conditions used during processing, in particular the intensity of milling and the temperature-time conditions of the malaxation [46,78]. Therefore, as already discussed, by choosing the appropriate agronomical and/or technological conditions it is feasible to produce VOO with a higher content in phenolic compounds [50,71]. Since some of these substances possess a bitter or pungent taste, the resulting oils may have a high intensity of these attributes [49]. Although bitterness and pungency are positive attributes in the official organoleptic evaluation of olive oil, when their magnitudes are excessive they may produce a decrease in the acceptance of the oil by the consumers.

However, when the enrichment or addition of antioxidants, biological active or aromatic functional ingredients from other raw materials are required or desired, different procedures must be used, mainly by infusion, extraction–enrichment or co-processing, as reported in Table 1. Since the olive oil regulations are very strict for the marketing of En-VOO, they must be denominated as “olive oil dressing/condiment with …” or “condiment produced using olives and …”.

***Infusion*.** Infusion, also known as maceration and solid–liquid extraction (SLE), has been used for a long time to flavouring different type of foods, alcoholic beverages as well as olive oil for example. This type of conventional aromatisation requires a long time to allow the extraction of the desired compounds, both flavours and/or bioactives, into the oily phase [72]. It is generally carried out under agitation at room temperature or warming up to 40–60 °C, for a period of time from several hours up to a few days [56] or even more than one month [51,62]. The final aromatised mixture needs to be filtered in order to remove traces of leaves or herbs from the oil. With this method the stability of the functional compounds is preserved and the healthy properties of the functional edible oils is improved.

The application of microwave [41] and more frequently of ultrasounds [32,40,64] accelerates the diffusion of the functional compounds into the olive oil. Ultrasound can be successfully applied to improve the flavouring operation of the olive oil as a result of the mechanical effect of cavitation, which speeds up the extraction of aromatic or bioactive compounds into the VOO. The processing time is therefore reduced from hours or days to few minutes when comparing to traditional maceration (i.e., using an intensity of ultrasounds of about 1 W/cm^2^ with a frequency of 25 kHz) [56].

A comparison of different mechanical methods (i.e., magnetic stirring, sonication and vertical stirring) for promoting the extraction of the antioxidants from oregano has been evaluated and the results obtained indicated that the best extraction procedure was vertical stirring at 1000 rpm. for 3 h [59].

***Extraction and enrichment*.** The protocol for the enrichment of edible oils by extraction involve two main steps: (i) the extraction of the target compounds from the raw material, for example, herbs or olive leaves; and then (ii) the enrichment of the oil with the obtained extract. The general procedure consisted in a liquid–solid extraction using ethanol, or ethanol: water (generally from 70:30 to 80:20 *v*/*v*); a little acidification (i.e., 0.1% formic acid) may also be required. Nevertheless, aqueous extracts of olive leaf and pomace, with or without the use of lecithin, in order to avoid the use of an organic solvent (ethanol or methanol) was also described [48].

As for the case of infusion, ultrasound or microwave irradiation (i.e., 8–10 min microwave irradiation at 200–400 W); can be used to accelerate the extraction process [42]; without applying ultrasound or microwave, a period of time from 12 to 24 h under agitation in dark may be necessary to perform the extraction [44]. After extraction, the suspension need to be centrifuged or filtered and finally concentrated several times or to dryness, in a rotary evaporator at a low temperature (35–40 °C) to avoid chemical alteration in the bioactive compounds with loss of their properties. The enrichment is then carried out by putting in contact an aliquot of the corresponding concentrated ethanolic extract with a certain amount of the oil; and the ethanol needs to be evaporated in the rotary evaporator at 30 °C. Finally, the mixture is shaken in an electrical stirrer to favour enrichment.

Another way to extract the essential oil from leaves, flowers or herbs is the use of hydrodistillation [58,60], employing a Clevenger-type apparatus to yield the corresponding essential oils (i.e., hydrodistillation for 2 h; with the oil then kept in dark flask at −18 °C).

An accelerated solvent extractor (i.e., ASE Dionex, Sunnyvale, CA, USA) allows faster extractions by using once again ethanol or methanol/water (i.e., 80:20, *v*/*v*) at high temperature and pressure (i.e., at 80 °C, up to 1500 psi) [36,42]. To improve the extraction of the desired compounds diatomaceous earth are combined with its source (i.e., at 1:2) to increase the contact surface and avoiding clogging of the cell. A few cycles of several minutes are used, each is programmed and then the extract is purged using nitrogen and rotary evaporated until all the solvent has been removed. Finally, it is freeze-dried. About 20% of phenolic extract from raw source material was obtained in these published studies [36,42].

Although rather expensive, SFE may be used to obtain the desired functional compounds [52]; supercritical CO_2_ conditions used in the cited work were an extraction time of 10 min, at a temperature of 40 °C, but applying different pressures (15–23 MPa) and superficial velocities (0.04–0.08 cm/s). Preparation of aromatised olive oil samples was performed by the addition of the extracts at a dose of 0.5% that were finally homogenised.

The dispersion of the bioactive extract and its stability in the En-VOO is of great relevance to guarantee the functional activity during its shelf life. For instance, when the effect of the addition of phenolic extracts from olive pomace and thyme was studied, results showed that flavonoids from thyme appeared to have higher transference ratios (89.7% as average) from the phenolic extract to oil, whereas secoiridoids from olive presented lower ratios (average 35.3%) [36]. On the other hand, it may be relevant to study the need for employing an emulsifier to promote the dispersion and the stabilization of the bioactive extract in the enriched product. A study indicated that lecithin is more convenient as an emulsifier than monoglyceride due to the higher oxidative stability obtained in the En-VOO [47]. In addition, the emulsifiers could mask the bitterness of the En-VOO, being preferable to improve the consumer preference of the new developed product.

With the purpose of yielding simple phenolics, Tyr and Htyr from their complex derivatives, an acid hydrolysis of olive leaf extract may be carried out (HCl 2 M, 100 °C for 1 h) [45]. The acid extract was then cooled and diluted with water and the hydrophobic fraction was extracted with ethyl acetate, which was subsequently removed by evaporation.

***Co-processing*.** A scarcely known innovative method is co-processing, based on the addition of herbs or other vegetable materials to the crushed olive paste before the malaxation step or during milling [63,68]; which may be implemented by the use of ultrasound before the olive paste malaxation [65]. In one of these studies, Cornicabra olives were co-processed under controlled conditions together with several fruits (apple, lemon and orange), spices (rosemary, thyme, basil and oregano) or leaves (rocket) in the Abencor laboratory system (Abengoa S.A., Seville, Spain) to obtain new VOO-based products [66,67,68]. The obtained results evidenced that the malaxation method was more effective in extracting the phenolic compounds, with a significantly lower level of hydrolysis of secoiridoids [63]. Moreover, when ultrasound was applied, the olive polyphenoloxidase—the endogenous enzyme responsible for olive oil phenol oxidation—was inhibited. As a consequence, antioxidant activity was significantly higher compared to the oils obtained by infusion [65]. Moreover, co-processing allows to produce oils ready to sell, instead the infused oils need to be filtered.

Co-processing olives with citrus fruits and/or zests gave rise to the presence of some new bioactive phenolics in the En-VOO, however, due to their polarity, citrus phenolics incorporated into the oil were not the major ones in the respective citrus fruits. Rancimat accelerated oxidation assay showed that the incorporation of orange and lemon during VOO processing produces a slight but not statistically significant increase in the oxidative stability of VOO-based products. On the other hand, it is interesting to note that citrus VOOs showed a DPPH antioxidant activity significantly higher than that of the control VOO, probably due to the contribution of the phenolic compounds coming from the citrus fruits analysed in this work [67].

## 4. Positive Outcomes in Prevention of Disease and Management of Health

Reactive oxygen species (ROS) are by-products of aerobic metabolism which include superoxide anion, hydrogen peroxide and hydroxyl radicals. Low or moderate concentrations of ROS are also involved in physiological responses as part of signalling processes and defence mechanisms against infectious agents [79]. An excessive production of ROS by exogenous redox chemicals, physical agents, bacterial or viral infections, or under abnormal pathophysiologic conditions induces a serious imbalance or mismatched redox equilibrium between production of ROS and the ability of cells to defend against them. This last situation is known as oxidative stress. Reactive oxygen species not only induce direct damage to critical biomolecules but also indirectly alter or dysregulate the cellular signalling events [80]. The pathophysiological consequences of such oxidative injury include cardiovascular and neurodegenative disorders, cancer, rheumatoid arthritis, etc. [80].

**Figure 1:**
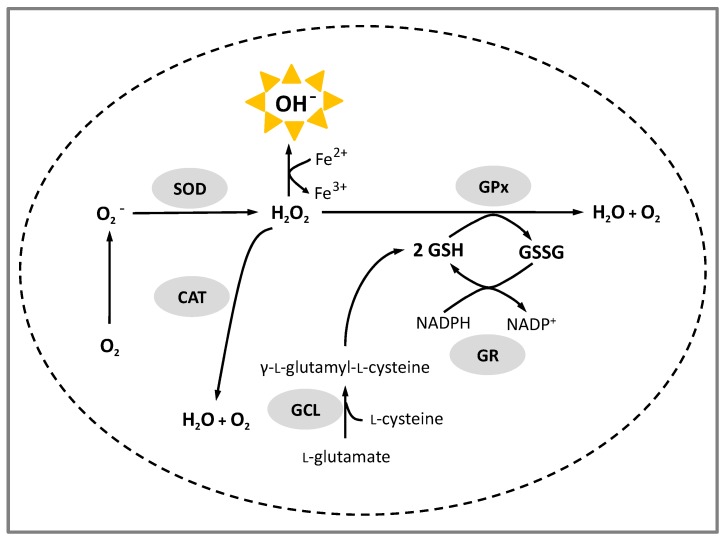
Cellular enzymes and mechanisms involved in protection from reactive oxygen species (ROS). SOD: Superoxide dismutase; CAT: Catalase; GPx: Glutathione peroxidase; GCL: Glutamate-cysteine ligase; GR: Glutathione reductase; GSH: Glutathione; GSSH: Oxidized glutathione; NADP^+^/NADPH: Nicotinamide adenine dinucleotide phosphate (oxidased/reduced).

The preservation of the redox status of the cell is vital for survival. Fortunately, cells are equipped with a cellular antioxidant defence system, composed of enzymatic and non-enzymatic components which can be classified into several categories: (i) metal chelators capable of preventing free radical formation by inhibiting metal catalysed reactions such as Fenton reaction; (ii) low-molecular weight antioxidants (e.g., glutathione (GSH) and ascorbic acid); (iii) enzymes synthesizing or regenerating the reduced forms of antioxidants such as glutamate-cysteine ligase (GCL) and glutathione reductase (GR); and (iv) ROS-interacting enzymes such as superoxide dismutases (SOD), glutathione peroxidases (GPx) and catalases (CAT) [81]. As can be seen in Figure 1, SOD inactivates superoxide anion by converting it to H_2_O_2_ which in turn is detoxified by CAT or GPx. While CAT reacts with H_2_O_2_ to form water and molecular oxygen, GPx detoxifies H_2_O_2_ producing water and oxidized glutathione (GSSG) which is recycled again to GSH by GR.

Over several decades, scientific studies have demonstrated that exogenous dietary phenolics provide cellular and tissue protection against oxidative damage [82]. Until now, phenolic effectiveness in providing health protection has been attributed to their antioxidant capacity (as a free radical scavenger mechanism) observed in vitro. However, kinetic limitations indicate that in vivo scavenging of radicals is ineffective in antioxidant defence. A recent theory supported that exogenous dietary phenolics generate signals for the induction of protective enzymes [82].

Multiple H_2_O_2_ sensors and pathways are triggered converging in the regulation of transcription factors, which induce the expression of a number of genes, including those required for the detoxification of oxidizing molecules and for the repair and maintenance of cellular homeostasis, controlling multiple cellular functions like cell proliferation, differentiation and apoptosis. The nuclear factor, erythroid 2-like 2 (Nrf2)– kelch-like ECH-associated protein 1 (Keap1) pathway is the major regulator of cytoprotective responses to oxidative and electrophilic stress [79].

Forman et al. [82] recently stated that phenolic compounds could be oxidised to electrophilic hydroquinones and quinones during their reaction with free radicals in vivo. In this way, quinones, even at low concentrations in vivo, appear to be critical to the ability to activate Nrf2 through Keap1 conjugation. These authors suggested two new terms: “nucleophilic tone” to describe the cellular, tissue, organ, or even organismal level of protection against electrophiles (including many free radicals and/or oxidants) by nucleophiles; and “para-hormesis” to describe the process by which non-toxic compounds (such as phenolic compounds) maintain an adaptive and defence system by mimicking electrophiles and increasing the nucleophilic tone.

After knowing the physiological mechanisms of action of nutritional phenolic compounds, it is necessary to study how En-VOOs could induce the signal transduction pathways to activate cellular antioxidant activities and damage/removal repair systems to confirm their healthful properties. To date, there are few publications focusing on functionality of En-VOO due to the novelty of this topic. Several works have provided the first results collected on positive effects of two En-VOO in appropriate human clinical trials. The first EnVOO—denominated FVOO, or functional virgin olive oil enriched with its phenolic compounds (500 ppm)—was obtained by adding a phenolic-rich extract obtained from the olive cake (oleuropein complex or secoiridoids: 89.4%; hydroxytyrosol, tyrosol and phenyl alcohols: 3.5%; and flavonoids: 6.0%) to an olive oil to reach 500 mg/L of phenolics [38]. The second EnVOO—FVOOT, or functional virgin olive enriched with its phenolic compounds (250 ppm) and those from thyme (250 ppm), was obtained by adding a phenol extract made up of a mixture of olive cake and dried thyme to an olive oil, thereby obtaining a flavouring olive oil rich in flavonoids (such as naringenin, eriodictyol and apigenin), phenolic acids (such as rosmarinic acid, ferulic acid and caffeic acid), and monoterpenes reaching 250 mg/L of phenolics from VOO and 250 mg/L of phenolics from thyme [36].

In these clinical trials, subjects were randomized to one of three orders of administration of raw olive oils: FVOO, FVOOT, VOO (sequence or intervention 1); FVOOT, VOO, FVOO (sequence or intervention 2); and VOO, FVOO, FVOOT (sequence or intervention 3); VOO with a moderate PC content (80 mg/L) was used as a control. In the crossover design, intervention periods were of three weeks with a daily ingestion of 25 mL of raw olive oils distributed along meals and preceded by two-week wash-out periods with a common olive oil [83]. Compliance was monitored through the determination of biomarkers of intake (hydroxytyrosolsulfate, hydroxytyrosol acetate sulfate, thymol sulfate and hydroxyphenylpropionic acid sulfate) analysing these phenolic metabolites in the subject’s biological fluids (urine and plasma), and a successful dietary intervention was guaranteed [34,84,85].

The main results of these clinical trials where enriched olive oils (FVOO and FVOOT) were administered are described as follows.

***Oxidative stress*.** Oxidative stress is characterized by an increased production of cellular oxidants that can attack lipid, protein and nucleic acid simultaneously in the living cells. Antioxidant endogenous enzymatic system shown in Figure 1 is responsible for protecting body cells against systemic oxidation [81].

Erythrocytes are particularly exposed to oxidative stress because they are oxygen carriers, have a high content of fatty acids and protein in their membranes and they have high haemoglobin, which can act as intracellular oxidative processes promoters. Erythrocytes, such as those described above are protected from oxidative injury via an antioxidant system composed among others of the enzymes SOD, CAT and GPx. Therefore, erythrocytes constitute a good experimental model for studying the mechanisms of injury from free radicals and antioxidant mechanisms. The effect of enriched olive oils (FVOO and FVOOT) on the antioxidant enzyme active improvement in hyperlipidemic subjects was studied by Romeu et al., 2016 [17]. In this case, a significant increase was registered in SOD activity 14-fold higher in FVOOT and two-fold higher to FVOO compared to VOO, respectively, attributed to the presence of polyphenol metabolites in erythrocytes. Therefore, olive oil PC can improve cellular protection, playing an important role in moderating the damage associated with ROS in situ. Identical results were described by Rubió et al., 2014 [84] when they evaluated the effect of the co-occurring components from olive oil and thyme extracts on the antioxidant status and its bioavailabilty in an acute ingestion in rats. Oliveras-López et al., 2014 [15] observed a positive association between a non-enriched Picual VOO (a rich-polyphenol oil with more than 700 mg/Kg) and plasma antioxidant capacity and, at the same time, a significant increase in CAT and GPx activities meanwhile, contrary to the previous authors, no significant difference was observed in SOD activity, perhaps due to the low levels of superoxide radical anions.

Romeu et al., 2016 [17] also assessed DNA protection against oxidation by the biomarker 8-hydroxy-2′-deoxyguanosine (8-OHdG) determination in urine samples; the 8-OHdG is the oxidized nucleoside of DNA most frequently detected and studied in DNA lesion. By other hand, the F2-isoprostane, 8-isoprostaglandin F2 α (8-iso PGF2α), provides a reliable tool for identifying populations with enhanced rates of lipid peroxidation. In plasma samples, the targets of oxidation in proteins were methionine (Met) residues, which are converted to methionine sulfoxide (MetSO). They demonstrated that the intake of FVOOT, which provides the same PC content but different PC profile than FVOO, produced the lowest 8-OHdG values and consequently appeared to improve DNA protection against oxidation rather than FVOO and VOO. The other biomarkers, 8-iso PGF2α (lipids) and MetSO (proteins), did not differ when the three VOO interventions were compared.

Based on the positive health effects of consumption of lycopene-enriched food, Garrido et al., 2013 [35] selected lycopene to produce En-VOOs. They evaluated the inclusion of lycopene to enhance the antioxidant properties produced by VOO. Lycopene, a bioactive red pigment, represents the most potent in vitro antioxidant among carotenoids [86]. They determined the urinary antioxidant capacity after its consumption and they compared it with the antioxidant capacity of VOO in young, middle-aged and elderly healthy subjects. The consumption of this enriched olive oil produced higher antioxidant effects in all of the three age groups assayed, being the lycopene circulating concentrations enough to combat oxidative stress and consequently prevent chronic diseases.

***Endothelial dysfunction and subsequent atherosclerosis and cardiovascular diseases*.** The main endothelial homeostatic functions involve: (i) regulation of vascular tone through a balanced production of vasodilatory and vasoconstrictor factors; (ii) maintenance of the blood fluidity and coagulation; and (iii) the production of cytokines and adhesion molecules that regulate inflammatory vascular function [87]. Among the compounds responsible for the vasodilatory capacity of endothelium, nitric oxide (NO) is probably the main endothelial-derived vasodilator. Clinical experience and prospective studies allow to establish a clear relation between oxidative stress as an early event in the development of endothelial dysfunction (ED) associated with a lower NO bioavailability [88]. It is also known that ED is a well-established response to cardiovascular risk factors (e.g., hypertriglyceridemia, hyperglycemia, hypertension blood) [89,90].

**Figure 2:**
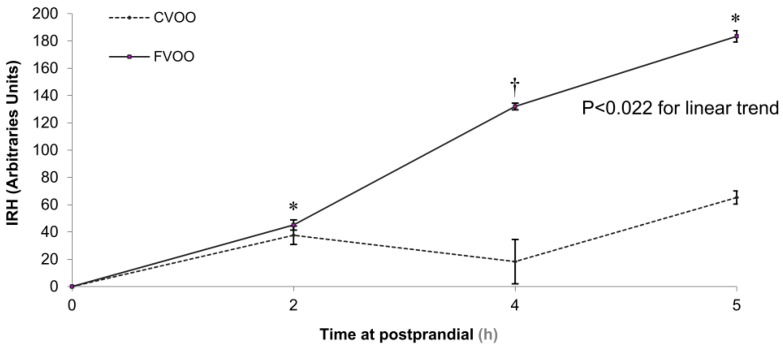
Postprandial time-course changes in ischemic reactive hyperemia (IRH) after ingestion of the different olive oils (OOs). CVOO: control virgin olive oil; FVOO: functional virgin olive oil enriched with its phenolic compounds (500 ppm). *, *P* < 0.05 versus baseline; †, *P* < 0.05 versus VOO at the same time-point. Figure reproduced with permission from Valls et al. [85].

In this sense, Valls et al., 2015 [85] have assessed the effects of FVOO intake on endothelial function in hypertensive patients. After collecting venous blood at the baseline (0 h) and at several time points after olive oil administration (2.5 and 5 h, considering these time intervals as postprandial state), the endothelial-dependent vasomotor function was determined by ischemic reactive hyperemia (IRH) and related biomarkers. As can be seen in Figure 2, these authors demonstrated that IRH increases in a linear trend after FVOO intake from baseline to 5 h, being this increase more than three- and four-fold at 4 h and 5 h respectively compared to 2 h postprandial. Hydroxytyrosol sulphate was the main quantitative biological metabolite and it increased in a dose-dependent manner with the polyphenol content of the olive oil administered (FVOO > VOO). At 4 and 5 h, the IRH for this enriched oil was also higher than those values obtained in the intervention control. As a consequence, En-VOO consumption improved the post-prandial endothelium-dependent microvascular dilatation in comparison with VOO. The authors also observed that oxidised low-density lipoprotein (LDL) decreased after FVOO ingestion in an inverse relationship with IRH values. According to these authors, this behaviour might be mediated via reduction in oxidative stress and the increase of NO metabolites during the post-prandial state as a consequence of the high-phenolic intake in plasma.

**Figure 3:**
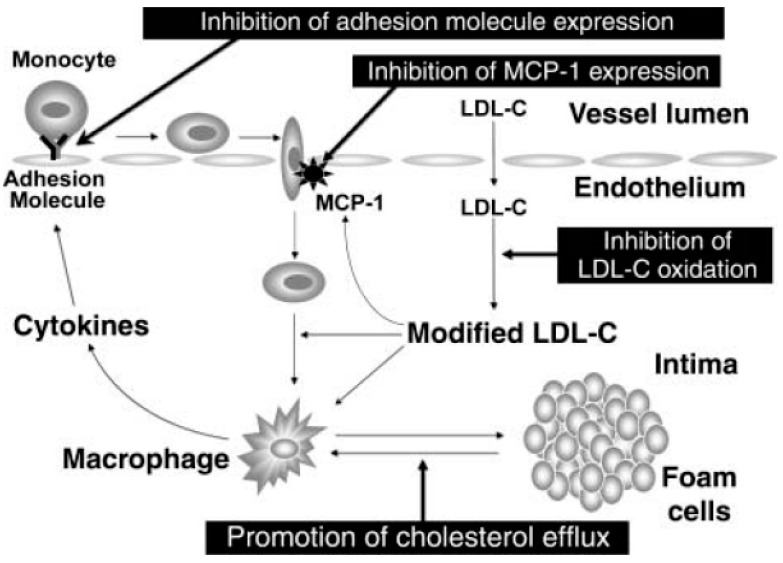
Role of low-density lipoproteins (LDLs) and early stages involved in atherosclerosis. Figure reproduced with permission from Barter [91]. LDL-C: LDL-cholesterol; MCP-1: Monocyte chemotactic protein-1.

Inflammatory processes have turned into a part of the pathophysiology of atherosclerosis and are involved from the initiation to the progression and final stages of infarction [13,91,92,93]. Some of the most important factors initiating atherosclerosis are LDLs. In Figure 3, Barter summarizes the role of LDLs in these early stages of atherosclerosis [91]. As can be observed, when LDL levels in plasma exceed a threshold, LDLs enter the artery faster than they can be removed, favouring their accumulation and modification, which includes oxidation processes. According to Barter, the modified LDLs stimulate endothelial cells to express a protein, monocyte chemotactic protein-1 (MCP-1), responsible for attracting monocytes from the blood into the artery wall [91]. In parallel form, the modified LDLs can promote the differentiation of monocytes into macrophages, which transform them in lipid-filled foam cells, the hallmark cells of atherosclerosis. The tumour necrosis factor-alpha (TNF-α) and interleukin (IL)-1 (both of which activate endothelial cells to express the adhesion molecules), E-selectin, P-selectin, vascular cell adhesion molecule-1 (VCAM-1), and intercellular adhesion molecule-1 (ICAM-1) are cytokines expressed by macrophages capable of binding plasma monocytes to the endothelium where they are attracted into the artery wall by MCP-1 and so on the cycle begins again, favouring atherosclerosis progression.

**Figure 4:**
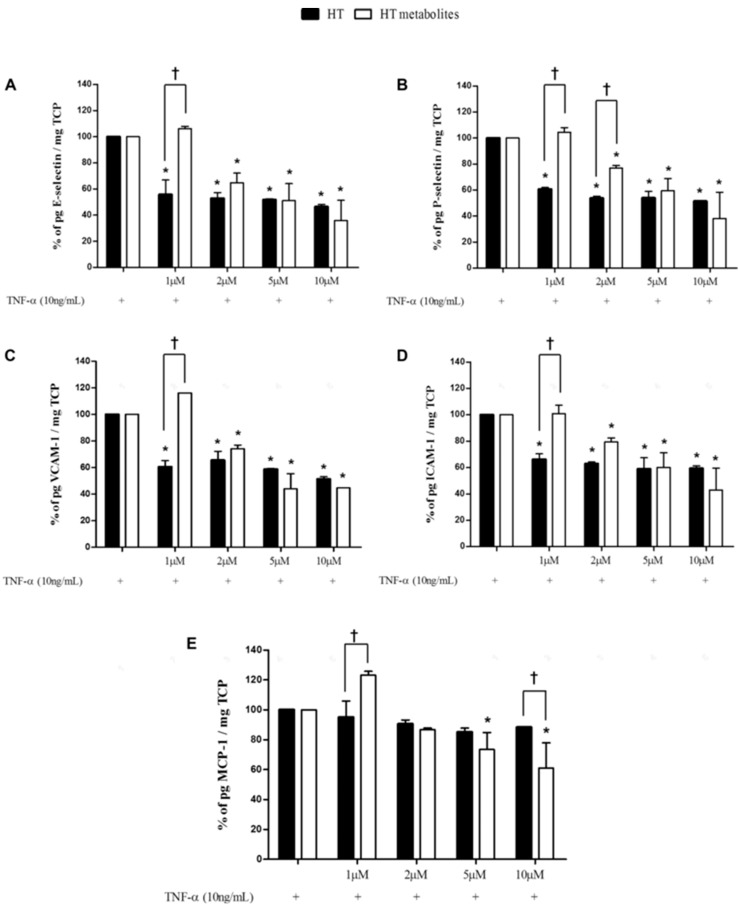
Effect of hydroxytyrosol (HTyr, or HT) and HT metabolites on E-selectin, P-selectin, VCAM-1, ICAM-1, and MCP-1 protein secretion in human aortic endothelial cells (HAEC) stimulated by TNF-α after 24 h. Human aortic endothelial cells were co-incubated with HT or HT metabolites at 1, 2, 5, and 10 μM and TNF-α (10 ng/mL) for 24 h. (**A**) Effect of HT or HT metabolites on E-selectin protein secretion. (**B**) Effect of HT or HT metabolites on P-selectin protein secretion. (**C**) Effect of HT or HT metabolites on VCAM-1 protein secretion. (**D**) Effect of HT or HT metabolites on ICAM-1 protein secretion. (**E**) Effect of HT or HT metabolites on MCP-1 protein secretion. Results are expressed as the percentage of soluble cellular adhesion molecules or chemokine protein secretion adjusted by total cellular protein and standard error of the mean (SEM; error bars). *, *P* < 0.05 versus TNF-α alone. †, *P* < 0.05 compared between HT and HT metabolites at the same concentration. Figure reproduced with permission from Catalán et al. [13]. TCP: Tissue culture plate; TNF-α: Tumour necrosis factor-alpha; VCAM-1: Vascular cell adhesion molecule-1; ICAM-1: Intercellular adhesion molecule-1; MCP-1: Monocyte chemotactic protein-1.

In order to demonstrate the olive phenolic protective role against the endothelial dysfunction, Catalán et al., 2015 [13] assessed in vitro the bioactivity of HTyr metabolites in human aortic endothelial cells (HAECs) co-incubated with TNF-α. Hydroxytyrosol is the main phenolic compound absorbed from the intestinal tract leading to the formation of phase II metabolites (sulfate, methyl, and methyl-sulfate conjugates and glucuronides) in the intestinal epithelium and the liver [84,94]. For this reason, they previously had to biosynthesize the main plasmatic HTyr metabolites through Caco-2 cells resulting HTyr sulfate as the main metabolite and homovanillic acid sulfate in minor proportion. By the other hand, it has been shown that TNF-α-induced increases in the expression of both E-selectin and VCAM-1 [91]. The authors selected E-selectin, P-selectin, VCAM-1, ICAM-1 and MCP-1 cytokines as endothelial dysfunction biomarkers related to the early stages of atherosclerosis. The results of this study confirmed that free HTyr and their metabolites were effective in the reduction of the selected ED biomarkers but only HTyr metabolites further reduced MCP-1 at 24 h, as can be seen in Figure 4.

**Figure 5:**
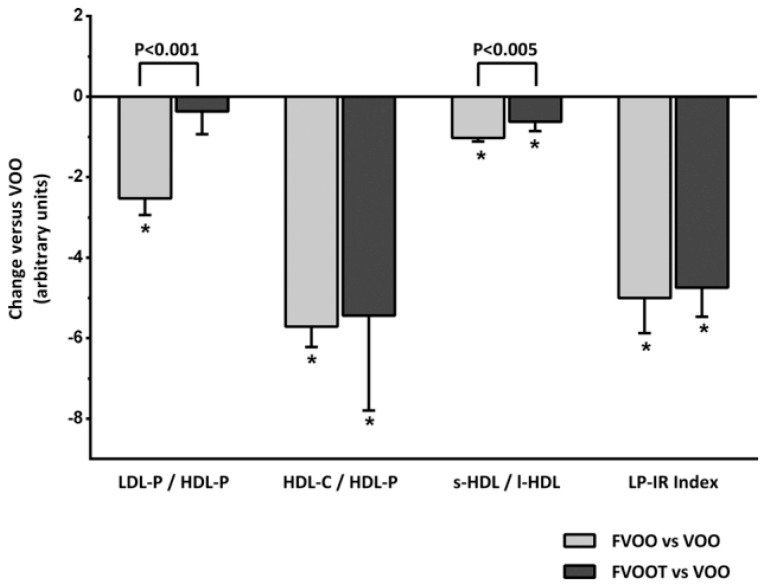
Changes in atherogenic lipoprotein particle atherogenic ratios and lipoprotein insulin resistance index (LP-IR) after consumption of functional olive oils versus natural virgin olive oil (VOO). FVOO: Functional virgin olive oil enriched with its phenolic compounds (500 ppm); FVOOT: Functional olive enriched with its phenolic compounds (250 ppm) and those from thyme (250 ppm). *, *P* < 0.001 versus VOO. Differences between functional olive oils are indicated by square brackets with the corresponding significance. Figure reproduced with permission from Fernández-Castillejo et al. [34]. HDL: high-density lipoprotein; HDL-C: HDL-cholesterol; LDL-P: low density lipoprotein-particles; HDL-P: HDL-particles; s-HDL: small HDL; l-HDL: large HDL.

High-density lipoproteins (HDLs) are regarded as the main anti-atherogenic and anti-thrombotic lipoproteins in the general population and increased levels of HDL (subfractions HDL3-C and particularly HDL2-C) have been associated with a protective effect against morbid cardiovascular events [91,95,96]. As has been commented, the EUROLIVE study contributed in the increment in HDL after a high polyphenol-olive oil intake in healthy volunteers. This advantageous effect of HDL is supposed to be accomplished primarily through the reverse cholesterol transport and the neutralization of oxidized lipids [97]. On the contrary, a proatherogenic lipid profile characteristic by high increased levels of total cholesterol, LDLs and triglycerides, and decreased levels of HDLs are important risk factors for cardiovascular diseases [95,98]. In this sense, Fernández-Castillejo et al., 2016 [34] determined the average particle size for the very low-density lipoprotein (VLDL), LDL and HDL groups by nuclear magnetic resonance (NMR) and their corresponding concentrations on serum samples after the intake of target En-VOOs on hypercholesterolemic individuals which are associated with the risk of coronary heart disease (CHD). Glucose and the classical cardiovascular lipid profile: total cholesterol; triglycerides, TG; LDL-cholesterol (LDL-C); and HDL-cholesterol (HDL-C) did not change with the consumption of the two assessed En-VOOs. However, changes in NMR lipoprotein particle counts and subclasses distribution were observed by these authors after En-VOO consumption, particularly after FVOO intake. By comparison with VOO consumption, they observed a decrease in the following parameters associated with the CHD risk: LDL-C concentrations, LDL-particles (LDL-P) and LDL-size after En-VOO consumption. On the other hand, FVOO and FVOOT promoted an increase in large HDL (l-HDL) and small HDL (s-HDL) particles, as well as a decrease in medium VLDL particles. Different lipoprotein ratios (LDL-P/HDL-particles (HDL-P), HDL-C/HDL-P and s-HDL/l-HDL) were established by the authors as a better marker for atherosclerosis risk than conventional lipid measures and, all were favourable to a dietary intervention with functional olive oils (see Figure 5). The LDL-P/HDL-P, a biomarker directly associated with risk of CHD, decreased after FVOO by decreasing LDL size; they are susceptible to oxidation or glycosilation, and as a consequence atherogenic potential is reduced. The biomarker HDL-C/HDL-P decreased by decreasing HDL-C which facilitates the efflux of cholesterol from peripheral cells. Finally, s-HDL/l-HDL ratio decreased; high levels of s-HDL or low levels of l-HDL are often related to CHD diseases.

**Figure 6:**
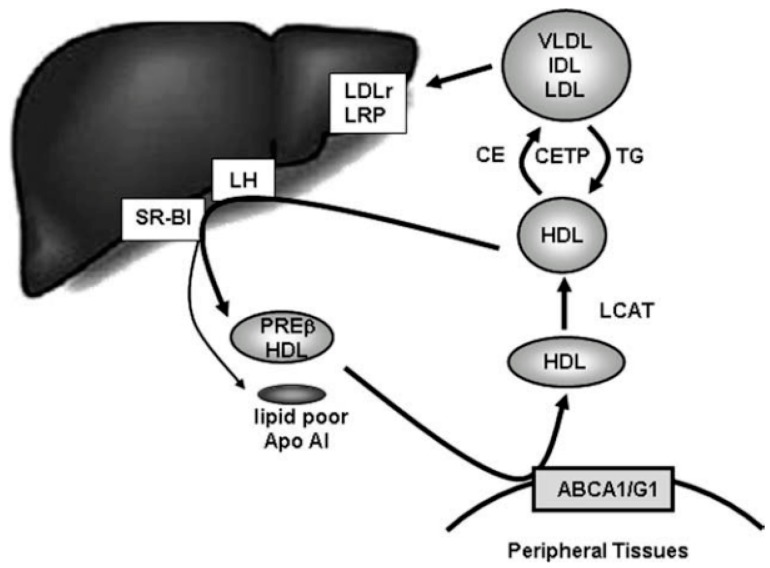
Stages involved in the reverse cholesterol transport (RCT), which favours the cholesterol transport to the liver for its excretion. Figure reproduced with permission from Oliveira and De Faria, 2011 [99]. LDLr: LDL receptors; LRP: LDL receptor-related proteins; CE: Cholesteryl ester; SR-BI: Scavenger receptor class B type I; VLDL: Very low-density lipoprotein; LH: Hepatic lipase; IDL: Intermediate-density lipoproteins; TG: Triglycerides; CETP: Cholesterylester transfer protein; LCAT: lecithin cholesterol acyl transferase; ABCA1/G1: ATP binding cassette transporter A1 or G1; Apo AI: Apolipoprotein AI.

As PC from olive oil has beneficial effects on lipid profile, Farràs et al., 2015 [83] proposed to study the effect of enriched oils on enhancing HDL functionality in hypercholesteraemic subjects associated with the risk of CHD. In Figure 6, the overview of lipoprotein metabolism with special reference to the role of reverse cholesterol transport (RCT) is presented [99]. According to Oliveira and De Faria, 2011 [99], in a first stage the ATP binding cassette transporter A1 or G1 (ABCA1/G1) membrane transporters interact with HDL subspecies to remove cholesterol from cell membranes. The cholesterol on the surface of HDL is esterified by the enzyme lecithin cholesterol acyl transferase (LCAT) which then enters into the hydrophobic core of the HDL particle. The HDL-cholesteryl ester (CE) can be delivered selectively to steroidogenic tissues (liver, adrenal, and gonadal) via scavenger receptor class B type I (SRBI) [98] but CE can be transferred to VLDL and LDL by the action of cholesterylester transfer protein (CETP) in exchange for TG [99]. Selective uptake of HDL-CE by SRBI and hydrolysis of HDL-TG by hepatic lipase (LH) contribute to (re)generation of prebeta-HDL and lipid poor apolipoprotein AI (Apo AI), which re-start the RCT cycle [99]. The LDL receptors (LDLr) and LDL receptor-related proteins (LRP) favour that the VLDL enriched in CE can be taken up mainly by the liver. Cholesterol in the liver can be secreted into the bile and excreted from the body via faeces [99].

Following this figure, Farràs et al., 2015 [83] observed increases in enzyme activities related with HDL metabolism and antioxidant status (LCAT and CETP) after FVOOT intervention, followed by FVOO, respect to the VOO control. As a consequence of these increases, LCAT in particular, an increase in HDL2-particle subclass percentage and a decrease in the HDL3-particles were registered after FVOOT intake, a favourable profile with a protective effect against cardiovascular mortality.

**Figure 7:**
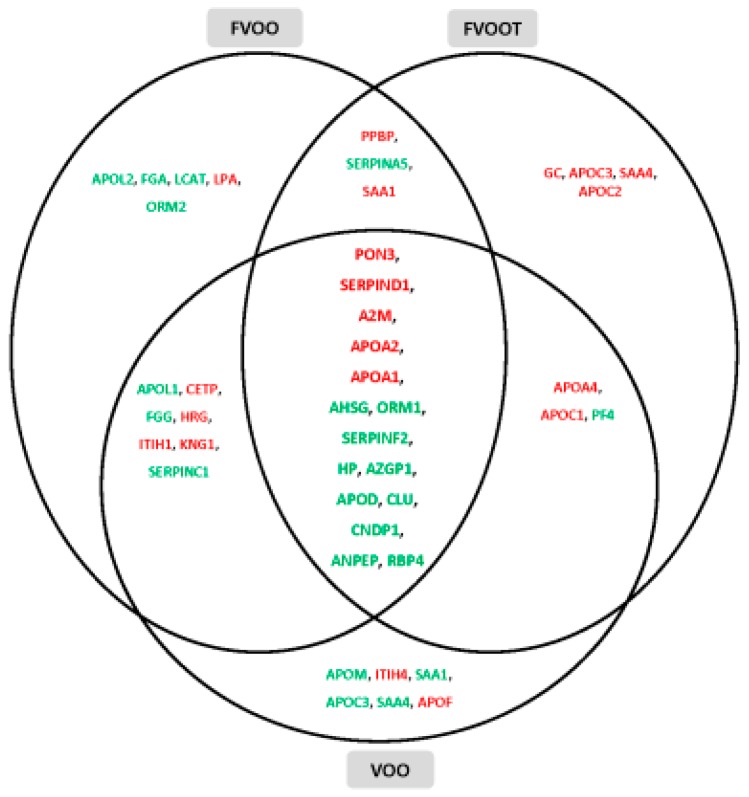
Venn diagram showing intersections of proteins differentially expressed after VOO, FVOO, and FVOOT interventions. Proteins are presented with their gene encode symbol. Red proteins: Up-regulated; Green proteins: Down-regulated. Figure reproduced with permission from Pedret et al. [100]. VOO: Virgin olive oil; FVOO: Functional virgin olive oil enriched with its own phenolic compounds; FVOOT: Functional virgin olive oil enriched with its own phenolic compounds plus complementary phenols from thyme.

The three VOO interventions changed the expression of a number of proteins with biological functions related to the cardio-protective function of the HDL particle [100]. According to these authors and, as can be seen in Figure 7, great expression modifications in 15 of the 127 proteins identified in the HDL fractions were produced, which were commonly up- or down-regulated and might lead to a cardiovascular disease (CVD)-protective HDL profile. The 15 proteins were mainly involved in liver X receptor/retinoid X receptor (LXR/RXR) activation acute phase response and atherosclerosis; this fact could be related to the capacity of transcriptional gene regulation of PC of VOOs [100].

***Intestinal immune function*.** It is well established that there is a relationship between healthy gut flora and health of the host due to their metabolic, immunological and gut protective functions [101]. The normal gut microbiota is involved in several functions [101] such as nutrient metabolism, xenobiotic and drug metabolism, antimicrobial protection, integrity of the gut barrier and structure of the gastrointestinal tract and development of gut-associated lymphoid tissue (GALT) which establishes the most extensive part of the immune system in the body. The crosstalk between GALT and the microbiota is critical for mucosal tissue homeostasis, maintenance of mucosal barrier function and protection against infectious and inflammatory diseases occurring at mucosal sites [102,103].

The ability of the normal intestinal microbiota to limit the integration of exogenously foreign bacteria into the existing population is referred to as colonization resistance [101,104]. As a consequence, the growth of intestinal pathogens in the intestinal lumen is prevented and the impact of disease is minimized. Anaerobic bacteria of the gut have been proposed to provide colonization resistance against pathogenic organisms such as *Salmonella* [101]; acetate and other short-chain fatty acids, products of obligate anaerobic metabolism, inhibit the growth of *Salmonella*. Recent studies characterised the ability of different strains of *Bifidobacterium* to provide protection against enterotoxigenic *Escherichia coli* infection. In this sense, Martín-Peláez et al., 2015 [16] investigated the effect on microbiota of those PC not absorbed by gastric and intestinal digestion processes which finally reach the gut and can serve as energy source for microbiota. An increase in HTyr and protocatechuic acid metabolites with antioxidant activities generated after gut microbial fermentation of En-VOOs was observed. It can be concluded that there is evidence of the potential prebiotic activity of an olive oil enriched in virgin olive oil and thyme PC by the specific growth stimulation of *Bifidobacteria* in the human gut.

Abnormal microbiota have been described in many inflammatory and autoimmune diseases (Crohn’s disease, ulcerative colitis, inflammatory arthritis, type-1 diabetes, multiple sclerosis, etc.). For this reason, Martín-Peláez et al., 2016 [103] evaluated the influence of En-VOO on human intestinal immune function in hypercholesterolemic participants establishing the following biomarkers in faecal samples: faecal immunoglobulin A (IgA) and IgA-coated bacteria as intestinal immunity biomarker; faecal protectin and cytokines—interleukin IL-6 and TNF-α—as intestinal inflammatory markers and plasma samples; and C-reactive protein (CRP) as a systemic inflammation marker. According to their results, the VOO intervention increased IgA-coated bacteria by 18.7%, followed by FVOO (51.6%), and FVOOT (94.3%) with respect to pre-intervention values (taking pre-values as 100%). For faecal IgA, a similar trend was found with increases around 59.7%, 52.8% and 205.3% for VOO, FVOO and FVOOT, respectively [103]. Faecal calprotectin is regularly used as indicator for the main inflammatory bowel diseases (IBD) such as Crohn’s disease and ulcerative colitis, meanwhile TNF-α and IL-6 are proinflammatory cytokines which have been linked with abdominal obesity and the metabolic syndrome. Although the three oil interventions tended to reduce these biomarkers, the decreases were not statistically significant and they only detected TNF-α for which there was no effect with oil interventions. In contrast, the results published by Fitó et al. [105] found that consumption of olive oil phenolic compounds from a daily dose of virgin olive oil decreased the circulating concentrations of both IL-6 and CRP in 28 stable coronary heart patients, Martín-Peláez et al., 2016 [103] registered an unexpected increase in CRP after En-VOO1 (500 mg/Kg), which could be due to the high PC consumption sustained for three weeks instead of acute ingestion, which resulted in a high accumulation of these bioactive compounds leading to undesirable effects. However, lower amounts of PC contained in VOO and/or higher amounts of PC contained in En-VOO2 resulting of the combination of two PC sources (olive oil and thyme) did not show significant effects on the investigated variables.

## 5. Future Directions and Opportunities in Oils Technology Research

***Future directions in En-VOO technology research***. The innovation in the olive sector is an increasingly reality. The production of enriched olive oil is opening new markets to the companies that have decided to make a leap of quality in the olive sector as a new strategy to enhance the beneficial effects of olive oil. An example is the case of Olive Oil Biotech (Jaen, Spain), a company that has just released its Walnutolive [106] oil, obtained from the co-crushing of olives and walnuts. Other trade initiative is the production of a VOO enriched with lycopene from tomato [107]. Nowadays, the consumers can also purchase a commercial VOO enriched with fish oil which provides a high content of omega 3 [108]. Finally, and within the marine field, a VOO flavoured with dehydrated seaweed has been released [109].

Limón et al. (2015) [110] demonstrated that carotenoids (β-carotene and lutein)-rich extracts from *Scenedesmus almeriensis* microalgae added to VOOs at different concentrations (0.1 and 0.21 mg/mL) improved the oxidative stability and, as a consequence, olive oil shelf-life and nutritional value. According to these authors, from the nutritional point of view the consumption of olive oils enriched with *S. almeriensis* extracts would be a valuable asset to counterbalance the deficient consumption of fruits and vegetables, reducing the lower ingestion of vital carotenoids. Regarding the beneficial effects on health, this study shows that VOOs enriched with lutein could be more effective in the prevention of degenerative diseases relating to vision.

Nevertheless, future works need to be carried out to assess the stability of this kind of olive oils during storage and during culinary process. In addition, after checking the positive effects of bioactive compounds in the enriched VOOs, scientific research must be focused on determining the exact amount that should be incorporated to make it beneficial.

***Future directions of En-VOO in nutrigenomic and nutrigenetic fields***. All the results presented so far confirm that food choices affect health and, therefore, consumers want to choose foods that are healthy for them. Personalized nutrition allows in practice adapting En-VOOs and other functional foods to individual needs in order to improve their quality of life due to consumers responding differently to the particular dietary components of these functional ingredients. However, the development of these functional foods is based, to a great extent, on empirical research rather than molecular nutrition [111].

Nutrigenomics—how dietary nutrients affect gene expression—and nutrigenetics—how genes affect nutrient metabolism- are indispensable tools for consumers to characterise the ‘dietary signature’, the relationship between their health and their nutritional status [112]. *Omics technologies* “are useful to assess all changes observed at the level of genes (*genomics and epigenomics*), gene transcripts (*transcriptomics*) and proteins as gene-encoded products (*proteomics*), as well as the intermediates and products of metabolism (*metabolomics*) which are comprised in these ‘dietary signature’” [113]. In this sense, Giordano et al. (2014) [114] published the first nutrigenomic study confirming that Htyr feeding, in nutritionally relevant amounts, modulates glutathione-mediated oxido-reduction pathways in adipose tissue. They also confirmed the GSH-modulating activities of HT in cultured adipocytes, where low, physiological HT concentrations were able to blunt the H_2_O_2_-induced GSH/GSSG alterations indicative of oxidative stress. In terms of surrogate markers of cardiovascular disease, authors recorded significantly decreased circulating leptin concentrations in mice fed with HT as compared with controls [114].

Scientific and technological advances should continue in understanding the regulation and variability of the human genome as a response to its exposure to the bioactive compounds (food genomes) in conjunction with the host’s gut microbiome. The future new insights into the interplay between genes and bioactive compounds from En-VOOs at the gene transcript, protein and metabolite levels will provide new biological markers. These new nutrigenomic associated biomarkers will be essential in monitoring the intake (exposure), molecular target (effect) and variation response (susceptibility) to develop a profile for an individual that reflects the effect of diet on overall performance and health.

## Figures and Tables

**Table 1:** Sources of natural bioactive ingredients and extraction conditions for the development of novel functional virgin olive oil (VOOs).

Source/Ingredient	Extraction Method and Conditions	Observed Functional Effect in Enriched Olive Oil	Reference
**Olive leaves**	SLE: directly in the oil (organic-solvent free), dynamic extraction chamber, USAE (25 °C, 20 min)	Increased oxidative stability	[40]
	SLE and LLE with ethanol, MAE (8–10 min, 200–400 W)	Increased oxidative stability	[41,42,43]
**Olive pomace**	SLE: ethanol/water (70:30), overnight stirring	Increased oxidative stability	[44]
	SLE: methanol and 1,2-propanediol, hydrolyzate (HCl 2 M, 100 °C for 1 h)	Increased phenolic content in fried foodstuffs	[45]
	LLE with ethyl acetate (for vegetative water) and SLE with ethanol/water (80:20; for solid residue)	Increased oxidative stability and antioxidant capacity	[38]
	ASE with ethanol/water (80:20 at 80 °C)	Increased oxidative stability and antioxidant capacity	[42]
	ASE with ethanol/water (80:20 at 80 °C), enrichment with 0.3% of emulsifier (lecithin or monoglycerides) and USAE	Lecithin is more effective increasing oxidative stability and antioxidant capacity	[47]
**Olive leaf and pomace**	Aqueous extracts with or without the use of lecithin	Increased oxidative stability	[48]
**Olive oil**	Semi-preparative HPLC	Increased antioxidant capacity	[49]
	Technological conditions during crushing (milling intensity) and malaxation (temperature and time)	Increased oxidative stability and antioxidant capacity	[50]
**Red pepper**	Infusion: 10%–20% up to 30 days	Reduced oxidative stability	[51]
	SFE at 40 °C and 15–23 MPa	Reduced oxidative stability	[52]
**Hot pepper, garlic, oregano and rosemary**	Infusion: 20–40 g/L up to 7 months	Increased oxidative stability	[53]
**Rosemary, lavender, sage, menthe, basil, lemon and thyme**	Infusion: 5% for 15 days	Increased oxidative stability for rosemary > thyme and > lemon	[54]
**Garlic, lemon, oregano, hot pepper, and rosemary**	Co-processing (pressing, crushing and malaxation): 3%–20% into olives	Reduced stability, expect garlic Higher antioxidant activity (rosemary)	[55]
**Basil**	Infusion: 15% with USAE (1 W/cm^2^) for 15 min	Not determined	[56]
**Lemon and thyme**	Infusion: 20% during 2 months	Unchanged oxidative stability	[57]
**Thyme and olive pomace**	ASE: ethanol/water (80:20) at 80 °C and up to 1500 psi	Increased antioxidant capacity	[36]
**Oregano**	Addition of 0.05% of essential oil	Increased oxidative stability	[58]
	Infusion by stirring at 1000 rpm for 3 h	Increased oxidative stability	[59]
**Thyme**	Addition of 200 mg/L of essential oil	Not clear effect on oxidative stability	[60]
**Garlic, hot chili peppers, laurel, oregano and pepper**	Infusion: 10 g/L during three months at room temperature	Increased oxidative stability	[61]
**Sweet lemon and sweet orange peels**	Infusion: 1%–5% at 60 °C for 40 days	Increased antioxidant capacity. Reduced oxidative stability	[62]
**Basil, chili and garlic**	Infusion: 10%–20%, 7 days stirring at 15–18 °C vs. combined malaxation	Antioxidant activity was significantly lower in the oils obtained by infusion	[63]
**Caraway**	1.5% at room temperature, infusion for 6 h vs. USAE for 30 min	Increased oxidative stability (OSI, but not for PV or Ks)	[64]
**Thyme and oregano**	Infusion (10 g/L for 15 days at room temperature) vs. co-malaxation (10 g/kg with or without 6 min USAE, before kneading)	Increased antioxidant capacity (especially with thyme)	[65]
**Citrus fruits or peel**	0.5%–5% by co-processing (milling and/or malaxation)	Increased antioxidant capacity	[66,67]
**Fruits (apple, lemon and orange), spices (rosemary, thyme, basil and oregano) and leaves (rocket)**	0.5%–5% by co-processing (milling and/or malaxation)	Statistically significant differences in sensory testing, except in apple and rocket	[68]
**Lycopene**	SLE with tomato pulp and VOO at high mechanical mixing (patented)	Enhanced antioxidant status in humans	[35,69]
	Co-milling of tomato seed or skin and olives	Significant enrichment in carotenoids, especially in lycopene	[70]

Oxidative stability or shelf-life, determined by peroxide value (PV), Ks or oxidative stability index (OSI/Rancimat). Antioxidant capacity, determined by 2,2-diphenyl-1-picrylhydrazyl (DPPH), oxygen radical antioxidant capacity (ORAC) or 2,2′-azino-bis(3-ethylbenzothiazoline-6-sulphonic acid) (ABTS) assays. ASE: Accelerated extraction system; HPLC: High-performance liquid chromatography; LLE: Liquid–liquid extraction; MAE: Microwave assisted extraction; SFE: Supercritical fluid extraction; SLE: Solid–liquid extraction; USAE: Ultrasound assisted extraction.

## Abbreviations

ABCA1/G1	ATP binding cassette transporter A1 or G1
ABTS	2,2′-azino-bis(3-ethylbenzothiazoline-6-sulphonic acid)
Apo AI	Apolipoprotein AI
ASE	Accelerated extraction system
CAT	Catalase
CE	Cholesteryl ester
CETP	Cholesteryl ester transfer protein
CHD	Coronary heart disease
CRP	C-reactive protein
CVD	Cardiovascular disease
DNA	Deoxyribonucleic acid
DPPH	2,2-diphenyl-1-picrylhydrazyl
ED	Endothelial dysfunction
EFSA	European Food Safety Authority
En-VOO	Enriched VOO
EVOO	Extra virgin olive oil
EUROLIVE	The Effect of Olive Oil on Oxidative Damage on European Populations
FOSHU	Foods for Specified Health Use
FVOO	Functional virgin olive oil enriched with its phenolic compounds (500 ppm)
FVOOT	Functional virgin olive enriched with its phenolic compounds (250 ppm) and those from thyme (250 ppm)
GALT	Gut-associated lymphoid tissue
GCL	Glutamate-cysteine ligase
GPx	Glutathione peroxidase
GR	Glutathione reductase
GSH	Glutathione
GSSG	Oxidized glutathione
HAEC	Human aortic endothelial cells
HDL	High-density lipoprotein
HDL-C	HDL-cholesterol
HDL-CE	High-density lipoprotein- cholesteryl ester
HDL-P	HDL-particles
HDL-TG	High-density lipoprotein- triglycerides
HPLC	High performance liquid chromatography
HTyr/HT	Hydroxytyrosol
IBD	Inflammatory bowel disease
ICAM-1	Intercellular adhesion molecule-1
IDL	Intermediate-density lipoproteins
IgA	Immunoglobulin A
IRH	Ischemic reactive hyperemia
8-iso PGF2α	F2-isoprostane, 8-isoprostaglandin
Keap1	Kelch-like ECH-associated protein 1
LCAT	Lecithin cholesterol acyl transferase
LDL	Low-density lipoprotein
LDL-C	LDL-cholesterol
LDL-P	LDL-particles
LDLr	LDL receptor
LH	Hepatic lipase
l-HDL	Large HDL
LLE	Liquid–liquid extraction
LP-IR	Lipoprotein insulin resistance index
LRP	LDL receptor related proteins
LXR	Liver X receptor
MAE	Microwave assisted extraction
MCFA	Medium chain fatty acids
MCP-1	Monocyte chemotactic protein-1
Met	Methionine
MetSO	Methionine sulfoxide
NMR	Nuclear magnetic resonance
NO	Nitric oxide
Nrf2	Nuclear factor, erythroid 2-like 2
8-OHdG	8-hydroxy-2′-deoxyguanosine
OO	Olive oil
ORAC	Oxygen radical antioxidant capacity‎
OSI	Oxidative stability index
PC	Phenolic compounds
PREDIMED	Prevention with Mediterranean diet
PV	Peroxide value
RCT	Reverse cholesterol transport
ROS	Reactive oxygen species
RXR	Retinoid X receptor
SEM	Standard error of the mean
SFE	Supercritical fluid extraction
SLE	Solid-liquid extraction
s-HDL	Small HDL
SOD	Superoxide dismutase
SRBI	Scavenger receptor class B type I
TC	Total cholesterol
TCP	Tissue culture plate
TG	Triglycerides
TNF-α	Tumour necrosis factor-α
Tyr	Tyrosol
USAE	Ultrasound assisted extraction
VCAM-1	Vascular cell adhesion molecule-1
VLDL	Very low-density lipoprotein
VOO	Virgin olive oil
